# Supplementary material for: Observational and genetic evidence highlight the association of human sleep behaviors with the incidence of fracture
Source: Commun Biol. 2021 Nov 26;4:1339. doi: 10.1038/s42003-021-02861-0 (PMC8626439; doi:10.1038/s42003-021-02861-0)
Supplement: Supplementary file 2 — Supplementary Information [file 42003_2021_2861_MOESM2_ESM.docx]

| **Supplementary Table 1** Baseline characteristics of participants who had and had not developed a fracture in prospective studies of fracture. | | |
| --- | --- | --- |
| Characteristic | Participants without fracture | Participants with fracture |
|  | (N = 385071) | (N = 13002) |
| Age^#^ | 56.49 (8.05) | 58.26 (7.75) |
| Sex^*^ |  |  |
| Female | 206683 (53.7) | 8163 (62.8) |
| Male | 178388 (46.3) | 4839 (37.2) |
| Education^*^ |  |  |
| No | 60827 (15.8) | 2470 (19.0) |
| Yes | 324244 (84.2) | 10532 (81.0) |
| Body mass index^#^ | 27.31 (4.71) | 27.08 (4.83) |
| Smoking^*^ |  |  |
| Never | 210936 (54.8) | 6891 (53.0) |
| Previous | 135893 (35.3) | 4613 (35.5) |
| Current | 38242 (9.9) | 1498 (11.5) |
| Alcohol consumption^*^ |  |  |
| Never | 11557 (3.0) | 471 (3.6) |
| Previous | 12238 (3.2) | 508 (3.9) |
| Current | 361276 (93.8) | 12023 (92.5) |
| Adequate physical activity^*^ |  |  |
| No | 11557 (3.0) | 471 (3.6) |
| Yes | 12238 (3.2) | 508 (3.9) |
| The cognitive impairment^*^ |  |  |
| No | 381707 (99.1) | 12480 (96.0) |
| Yes | 3364 (0.9) | 522 (4.0) |
| The status of depression^*^ |  |  |
| No | 381701 (99.1) | 12479 (96.0) |
| Yes | 3370 (0.9) | 523 (4.0) |
| The use of glucocorticoid^*^ |  |  |
| No | 340139 (88.3) | 11477 (88.3) |
| Yes | 44932 (11.7) | 1525 (11.7) |
| The use of sleep medicine^*^ |  |  |
| No | 375723 (97.6) | 12548 (96.5) |
| Yes | 9348 (2.4) | 454 (3.5) |
| ^*^Values are numbers (percentages); | | |
| ^#^Values are mean (standard deviation); | | |
| Abbreviations: N, number. | | |

| **Supplementary Table 2** Assessment of the mediator (i.e., falls) for the association between insomnia and fracture risk^*^. | | | | |
| --- | --- | --- | --- | --- |
|  | Estimate | 95% CI Lower | 95% CI Upper | *P*-value |
| ACME (control) | 4.07E-04 | 3.64E-04 | 0.00 | <2E-16 |
| ACME (treated) | 4.25E-04 | 3.83E-04 | 0.00 | <2E-16 |
| ADE (control) | 0.001 | 0.001 | 0.00 | <2E-16 |
| ADE (treated) | 0.001 | 0.001 | 0.00 | <2E-16 |
| Total Effect | 0.002 | 0.001 | 0.00 | <2E-16 |
| Prop. Mediated (control) | 0.242 | 0.175 | 0.31 | <2E-16 |
| Prop. Mediated (treated) | 0.251 | 0.185 | 0.32 | <2E-16 |
| ACME (average) | 4.16E-04 | 3.75E-04 | 0.00 | <2E-16 |
| ADE (average) | 0.001 | 0.001 | 0.00 | <2E-16 |
| Prop. Mediated (average) | 0.246 | 0.180 | 0.31 | <2E-16 |
| ^*^ This mediation analysis was adjusted for age, sex, body mass index, education, smoking, alcohol consumption, physical activity, cognitive impairment, depression, and the use of glucocorticoid medication, benzodiazepines, and antidepressants. Abbreviations: ACME, average causal mediation effect; ADE, average direct effect; CI, confidence interval. | | | | |

| **Supplementary Table 3** Assessment of the mediator (i.e., bone mineral density) for the association between insomnia and fracture risk^*^. | | | | |
| --- | --- | --- | --- | --- |
|  | Estimate | 95% CI Lower | 95% CI Upper | *P*-value |
| ACME (control) | 2.00E-04 | 1.67E-04 | 0.00 | <2E-16 |
| ACME (treated) | 2.11E-04 | 1.77E-04 | 0.00 | <2E-16 |
| ADE (control) | 0.002 | 0.001 | 0.00 | <2E-16 |
| ADE (treated) | 0.002 | 0.001 | 0.00 | <2E-16 |
| Total Effect | 0.002 | 0.001 | 0.00 | <2E-16 |
| Prop. Mediated (control) | 0.104 | 0.074 | 0.17 | <2E-16 |
| Prop. Mediated (treated) | 0.110 | 0.079 | 0.17 | <2E-16 |
| ACME (average) | 2.05E-04 | 1.73E-04 | 0.00 | <2E-16 |
| ADE (average) | 0.002 | 0.001 | 0.00 | <2E-16 |
| Prop. Mediated (average) | 0.107 | 0.077 | 0.17 | <2E-16 |
| ^*^ This mediation analysis was adjusted for age, education, sex, smoking, alcohol consumption, physical activity, body mass index, and the use of glucocorticoid. Abbreviations: ACME, average causal mediation effect; ADE, average direct effect; CI, confidence interval. | | | | |

| **Supplementary Table 4** Assessment of the mediator (i.e., falls) for the association between sleep duration and fracture risk^*^. | | | | |
| --- | --- | --- | --- | --- |
|  | Estimate | 95% CI Lower | 95% CI Upper | *P*-value |
| ACME (control) | -2.69E-04 | -3.34E-04 | 0.00 | <2E-16 |
| ACME (treated) | -2.61E-04 | -3.24E-04 | 0.00 | <2E-16 |
| ADE (control) | -0.001 | -0.002 | 0.00 | <2E-16 |
| ADE (treated) | -0.001 | -0.002 | 0.00 | <2E-16 |
| Total Effect | -0.001 | -0.002 | 0.00 | <2E-16 |
| Prop. Mediated (control) | 0.192 | 0.122 | 0.30 | <2E-16 |
| Prop. Mediated (treated) | 0.188 | 0.118 | 0.30 | <2E-16 |
| ACME (average) | -2.65E-04 | -3.29E-04 | 0.00 | <2E-16 |
| ADE (average) | -0.001 | -0.002 | 0.00 | <2E-16 |
| Prop. Mediated (average) | 0.190 | 0.120 | 0.30 | <2E-16 |
| ^*^ This mediation analysis was adjusted for age, sex, body mass index, education, smoking, alcohol consumption, physical activity, cognitive impairment, depression, and the use of glucocorticoid medication, benzodiazepines, and antidepressants. Abbreviations: ACME, average causal mediation effect; ADE, average direct effect; CI, confidence interval. | | | | |

| **Supplementary Table 5** Assessment of the mediator (i.e., bone mineral density) for the association between sleep duration and fracture risk^*^. | | | | |
| --- | --- | --- | --- | --- |
|  | Estimate | 95% CI Lower | 95% CI Upper | *P*-value |
| ACME (control) | -1.88E-04 | -2.41E-04 | 0.00 | <2E-16 |
| ACME (treated) | -1.82E-04 | -2.31E-04 | 0.00 | <2E-16 |
| ADE (control) | -0.001 | -0.002 | 0.00 | <2E-16 |
| ADE (treated) | -0.001 | -0.002 | 0.00 | <2E-16 |
| Total Effect | -0.001 | -0.002 | 0.00 | <2E-16 |
| Prop. Mediated (control) | 0.129 | 0.086 | 0.23 | <2E-16 |
| Prop. Mediated (treated) | 0.125 | 0.082 | 0.23 | <2E-16 |
| ACME (average) | -1.85E-04 | -2.36E-04 | 0.00 | <2E-16 |
| ADE (average) | -0.001 | -0.002 | 0.00 | <2E-16 |
| Prop. Mediated (average) | 0.127 | 0.084 | 0.23 | <2E-16 |
| ^*^ This mediation analysis was adjusted for age, sex, body mass index, education, smoking, alcohol consumption, physical activity, cognitive impairment, depression, and the use of glucocorticoid medication, benzodiazepines, and antidepressants. Abbreviations: ACME, average causal mediation effect; ADE, average direct effect; CI, confidence interval. | | | | |

| **Supplementary Table 6** Association of sleep duration with fracture risk in male and female in observational analyses. | | | |
| --- | --- | --- | --- |
| Method | OR/HR | 95% CI | *P-*value of |
|  |  |  | association |
| Multivariable Cox regression in male (model 0) |  |  |  |
| sleep 7-8 hours | 1 (reference) | 1 (reference) | \ |
| sleep less 7 hours | 1.173 | 1.099-1.252 | 1.70×10^-6^ |
| sleep more than 8 hours | 1.200 | 1.079-1.334 | 7.74×10^-4^ |
| Multivariable Cox regression in male (model 1) |  |  |  |
| sleep 7-8 hours | 1 (reference) | 1 (reference) | \ |
| sleep less 7 hours | 1.155 | 1.081-1.234 | 1.88×10^-5^ |
| sleep more than 8 hours | 1.166 | 1.047-1.299 | 0.005 |
| Multivariable Cox regression in male (model 2) |  |  |  |
| sleep 7-8 hours | 1 (reference) | 1 (reference) | \ |
| sleep less 7 hours | 1.138 | 1.065-1.215 | 1.19×10^-4^ |
| sleep more than 8 hours | 1.159 | 1.042-1.289 | 0.007 |
| Multivariable Cox regression in male (model 3) |  |  |  |
| sleep 7-8 hours | 1 (reference) | 1 (reference) | \ |
| sleep less 7 hours | 1.121 | 1.049-1.198 | 7.44×10^-4^ |
| sleep more than 8 hours | 1.125 | 1.010-1.254 | 0.033 |
| Multivariable Cox regression in female (model 0) |  |  |  |
| sleep 7-8 hours | 1 (reference) | 1 (reference) | \ |
| sleep less 7 hours | 1.110 | 1.055-1.168 | 6.31×10^-5^ |
| sleep more than 8 hours | 1.081 | 0.997-1.171 | 0.059 |
| Multivariable Cox regression in female (model 1) |  |  |  |
| sleep 7-8 hours | 1 (reference) | 1 (reference) | \ |
| sleep less 7 hours | 1.098 | 1.043-1.156 | 3.74×10^-4^ |
| sleep more than 8 hours | 1.072 | 0.988-1.162 | 0.095 |
| Multivariable Cox regression in female (model 2) |  |  |  |
| sleep 7-8 hours | 1 (reference) | 1 (reference) | \ |
| sleep less 7 hours | 1.085 | 1.031-1.142 | 0.002 |
| sleep more than 8 hours | 1.064 | 0.982-1.154 | 0.130 |
| Multivariable Cox regression in female (model 3) |  |  |  |
| sleep 7-8 hours | 1 (reference) | 1 (reference) | \ |
| sleep less 7 hours | 1.075 | 1.021-1.132 | 0.006 |
| sleep more than 8 hours | 1.054 | 0.972-1.143 | 0.204 |
| Model 0 was adjusted for confounders, including age, sex, body mass index, education, smoking, alcohol consumption, physical activity, cognitive impairment, depression, and the use of glucocorticoid medication, benzodiazepines, and antidepressants; Model 1 = Model 0 + BMD; Model 2 = Model 0 +falls; Model 3 = Model 0+ BMD + falls. * the *P*-value of the intercept term. | | | |
| Abbreviations: BMD, bone mineral density; CI, confidence interval; HR, hazard ratio; IVW, inverse-variance weighted; MR, Mendelian randomization; MR-PRESSO, MR pleiotropy residual sum and outlier; OR, odds ratio. | | | |

| **Supplementary Table 7** Association of snoring with fracture risk in observational and Mendelian randomization analyses. | | | |
| --- | --- | --- | --- |
| Method | OR/HR | 95% CI | *P*-value of |
|  |  |  | association |
| Observational study |  |  |  |
| Multivariable Cox regression (model 0) | 0.951 | 0.914-0.989 | 0.012 |
| Multivariable Cox regression (model 1) | 0.942 | 0.905-0.980 | 0.003 |
| Multivariable Cox regression (model 2) | 0.947 | 0.911-0.985 | 0.007 |
| Multivariable Cox regression (model 3) | 0.939 | 0.902-0.977 | 0.002 |
| One-sample mendelian randomization (model 1) | 2.134 | 0.932-4.887 | 0.073 |
| Two-sample Mendelian randomization |  |  |  |
| IVW method | 1.214 | 0.770-1.913 | 0.402 |
| Weighted-median method | 0.861 | 0.517-1.433 | 0.564 |
| MR-PRESSO Outlier corrected | 1.029 | 0.722-1.466 | 0.875 |
| MR-Egger regression | \ | \ | 0.303* |
| Observational study |  |  |  |
| Multivariable Cox regression in male (model 0) | 0.870 | 0.820-0.924 | 5.73×10^-6^ |
| Multivariable Cox regression in male (model 1) | 0.865 | 0.814-0.919 | 2.61×10^-6^ |
| Multivariable Cox regression in male (model 2) | 0.874 | 0.823-0.928 | 1.02×10^-5^ |
| Multivariable Cox regression in male (model 3) | 0.869 | 0.818-0.923 | 5.54×10^-6^ |
| Multivariable Cox regression in female (model 0) | 1.018 | 0.967-1.072 | 0.503 |
| Multivariable Cox regression in female (model 1) | 1.003 | 0.953-1.057 | 0.902 |
| Multivariable Cox regression in female (model 2) | 1.009 | 0.958-1.062 | 0.740 |
| Multivariable Cox regression in female (model 3) | 0.995 | 0.945-1.049 | 0.862 |
| Multivariable Cox regression in participants without insomnia (model 0) | 0.994 | 0.915-1.081 | 0.890 |
| Multivariable Cox regression in participants with normal sleep duration (model 0) | 0.989 | 0.942-1.038 | 0.643 |
| Multivariable Cox regression in participants with non-insomnia and normal sleep duration (model 0) | 1.063 | 0.965-1.171 | 0.218 |
| Model 0 was adjusted for confounders, including age, sex, body mass index, education, smoking, alcohol consumption, physical activity, cognitive impairment, depression, and the use of glucocorticoid medication, benzodiazepines, and antidepressants; Model 1 = Model 0 + BMD; Model 2 = Model 0 +falls; Model 3 = Model 0+ BMD + falls. | | | |
| * the *P*-value of the intercept term. |  |  |  |
| Abbreviations: BMD, bone mineral density; CI, confidence interval; HR, hazard ratio; IVW, inverse-variance weighted; MR, Mendelian randomization; MR-PRESSO, MR pleiotropy residual sum and outlier; OR, odds ratio. | | | |

| **Supplementary Table 8** Baseline characteristics of participants with snoring and non-snoring. | | |
| --- | --- | --- |
| Baseline characteristics | Snoring | |
|  | No | Yes |
| Number of participants | 205957 | 121621 |
| Sleep duration^a^ | 7.16 (1.06) | 7.21 (1.06) |
| Chronotype^b^ |  |  |
| Morning perference | 188562 (91.6) | 110545 (90.9) |
| Evening perference | 17395 (8.4) | 11076 (9.1) |
| Insomnia^b^ |  |  |
| No | 48652 (23.6) | 32262 (26.5) |
| Yes | 157305 (76.4) | 89359 (73.5) |
| Excessive daytime sleepiness^b^ |  |  |
| No | 165130 (80.2) | 89018 (73.2) |
| Yes | 40827 (19.8) | 32603 (26.8) |
| ^a^ values are mean (SD); ^b^ values are numbers (percentages). | | |

| **Supplementary Table 9** Association of chronotype and daytime sleepiness with fracture risk in observational and Mendelian randomization analyses. | | | |
| --- | --- | --- | --- |
| Method | OR/HR | 95% CI | *P*-value of |
|  |  |  | association |
| ***Excessive daytime sleepiness and fracture*** |  |  |  |
| Observational study |  |  |  |
| Multivariable Cox regression (model 0) | 1.076 | 1.039-1.113 | 3.20×10^-5^ |
| Multivariable Cox regression (model 1) | 1.064 | 1.028-1.102 | 4.31×10^-4^ |
| Multivariable Cox regression (model 2) | 1.042 | 1.006-1.078 | 0.020 |
| Multivariable Cox regression (model 3) | 1.032 | 0.997-1.069 | 0.073 |
| Multivariable Cox regression in male (model 0) | 1.113 | 1.054-1.174 | 1.08×10^-4^ |
| Multivariable Cox regression in male (model 1) | 1.103 | 1.044-1.165 | 4.50×10^-4^ |
| Multivariable Cox regression in male (model 2) | 1.073 | 1.016-1.132 | 0.011 |
| Multivariable Cox regression in male (model 3) | 1.064 | 1.007-1.124 | 0.028 |
| Multivariable Cox regression in female (model 0) | 1.059 | 1.013-1.107 | 0.012 |
| Multivariable Cox regression in female (model 1) | 1.046 | 0.999-1.094 | 0.051 |
| Multivariable Cox regression in female (model 2) | 1.029 | 0.984-1.076 | 0.207 |
| Multivariable Cox regression in female (model 3) | 1.018 | 0.973-1.065 | 0.431 |
| ***Chronotype and fracture*** |  |  |  |
| Observational study |  |  |  |
| Multivariable Cox regression (model 0) | 0.963 | 0.944-0.982 | 1.79×10^-4^ |
| Multivariable Cox regression (model 1) | 0.970 | 0.951-0.989 | 0.002 |
| Multivariable Cox regression (model 2) | 0.966 | 0.947-0.985 | 4.00×10^-4^ |
| Multivariable Cox regression (model 3) | 0.972 | 0.953-0.992 | 0.005 |
| One-sample Mendelian randomization | 0.979 | 0.916-1.047 | 0.538 |
| Two-sample Mendelian randomization |  |  |  |
| IVW method | 0.986 | 0.954-1.020 | 0.425 |
| Weighted-median method | 1.003 | 0.959-1.049 | 0.910 |
| MR-PRESSO Outlier corrected | 0.996 | 0.965-1.029 | 0.814 |
| MR-Egger regression | \ | \ | 0.623* |
| Observational study |  |  |  |
| Multivariable Cox regression in male (model 0) | 0.957 | 0.926-0.989 | 0.008 |
| Multivariable Cox regression in male (model 1) | 0.960 | 0.929-0.993 | 0.016 |
| Multivariable Cox regression in male (model 2) | 0.959 | 0.929-0.991 | 0.012 |
| Multivariable Cox regression in male (model 3) | 0.963 | 0.932-0.995 | 0.024 |
| Multivariable Cox regression in female (model 0) | 0.971 | 0.947-0.995 | 0.018 |
| Multivariable Cox regression in female (model 1) | 0.978 | 0.954-1.003 | 0.079 |
| Multivariable Cox regression in female (model 2) | 0.973 | 0.949-0.997 | 0.029 |
| Multivariable Cox regression in female (model 3) | 0.980 | 0.956-1.005 | 0.114 |
| Model 0 was adjusted for confounders, including age, sex, body mass index, education, smoking, alcohol consumption, physical activity, cognitive impairment, depression, and the use of glucocorticoid medication, benzodiazepines, and antidepressants; Model 1 = Model 0 + BMD; Model 2 = Model 0 +falls; Model 3 = Model 0+ BMD + falls. | | | |
| * the *P*-value of the intercept term. |  |  |  |
| Abbreviations: BMD, bone mineral density; CI, confidence interval; HR, hazard ratio; IVW, inverse-variance weighted; MR, Mendelian randomization; MR-PRESSO, MR pleiotropy residual sum and outlier; OR, odds ratio. | | | |

| **Supplementary Table 10** Assessment of the mediator (i.e., falls) for the association between sleep risk score and fracture risk^*^. | | | | |
| --- | --- | --- | --- | --- |
|  | Estimate | 95% CI Lower | 95% CI Upper | *P*-value |
| ACME (control) | 4.22E-04 | 3.75E-04 | 0.00 | <2E-16 |
| ACME (treated) | 4.49E-04 | 3.94E-04 | 0.00 | <2E-16 |
| ADE (control) | 0.002 | 0.001 | 0.00 | <2E-16 |
| ADE (treated) | 0.002 | 0.001 | 0.00 | <2E-16 |
| Total Effect | 0.002 | 0.002 | 0.00 | <2E-16 |
| Prop. Mediated (control) | 0.177 | 0.134 | 0.24 | <2E-16 |
| Prop. Mediated (treated) | 0.188 | 0.146 | 0.25 | <2E-16 |
| ACME (average) | 4.36E-04 | 3.85E-04 | 0.00 | <2E-16 |
| ADE (average) | 0.002 | 0.001 | 0.00 | <2E-16 |
| Prop. Mediated (average) | 0.182 | 0.140 | 0.24 | <2E-16 |
| ^*^ This mediation analysis was adjusted for age, sex, body mass index, education, smoking, alcohol consumption, physical activity, cognitive impairment, depression, and the use of glucocorticoid medication, benzodiazepines, and antidepressants. Abbreviations: ACME, average causal mediation effect; ADE, average direct effect; CI, confidence interval. | | | | |

| **Supplementary Table 11** Assessment of the mediator (i.e., bone mineral density) for the association between sleep risk score and fracture risk^*^. | | | | |
| --- | --- | --- | --- | --- |
|  | Estimate | 95% CI Lower | 95% CI Upper | *P*-value |
| ACME (control) | 2.92E-04 | 2.50E-04 | 0.00 | <2E-16 |
| ACME (treated) | 3.13E-04 | 2.71E-04 | 0.00 | <2E-16 |
| ADE (control) | 0.002 | 0.002 | 0.00 | <2E-16 |
| ADE (treated) | 0.002 | 0.002 | 0.00 | <2E-16 |
| Total Effect | 0.002 | 0.002 | 0.00 | <2E-16 |
| Prop. Mediated (control) | 0.118 | 0.088 | 0.15 | <2E-16 |
| Prop. Mediated (treated) | 0.126 | 0.097 | 0.16 | <2E-16 |
| ACME (average) | 3.03E-04 | 2.60E-04 | 0.00 | <2E-16 |
| ADE (average) | 0.002 | 0.002 | 0.00 | <2E-16 |
| Prop. Mediated (average) | 0.122 | 0.092 | 0.15 | <2E-16 |
| ^*^ This mediation analysis was adjusted for age, sex, body mass index, education, smoking, alcohol consumption, physical activity, cognitive impairment, depression, and the use of glucocorticoid medication, benzodiazepines, and antidepressants. Abbreviations: ACME, average causal mediation effect; ADE, average direct effect; CI, confidence interval. | | | | |

| **Supplementary Table 12** Detailed information on the genome-wide association studies for four sleep behaviors (chronotype, insomnia, snoring, and sleep duration) and summary statistics of fracture, falls and bone miner density. | | | | |
| --- | --- | --- | --- | --- |
| Phenotype | Sample size | Ancestry | Links for downloading summary-level data | PMID |
| Exposure |  |  |  |  |
| Chronotype | 697,828 individuals | European ancestry | - | 30696823 |
| Insomnia | 1,331,010 individuals | European ancestry | - | 30804565 |
| Sleep duration | 446,118 individuals | European ancestry | - | 30846698 |
| Snoring | 408,317 individuals  (number of cases ≈ 152,000; number of controls ≈ 256,000) | European ancestry | - | 32060260 |
| Outcome |  |  |  |  |
| Bone mineral density | 426,824 individuals | Caucasian British | <http://www.gefos.org/?q=content/data-release-2018> | 30598549 |
| Falls | 361,194 individuals | \ | www.nealelab.is/uk-biobank/ | - |
| Fracture | 426,795 individuals  (number of cases = 53,184; number of controls = 373,611) | Caucasian British | <http://www.gefos.org/?q=content/data-release-2018> | 30598549 |


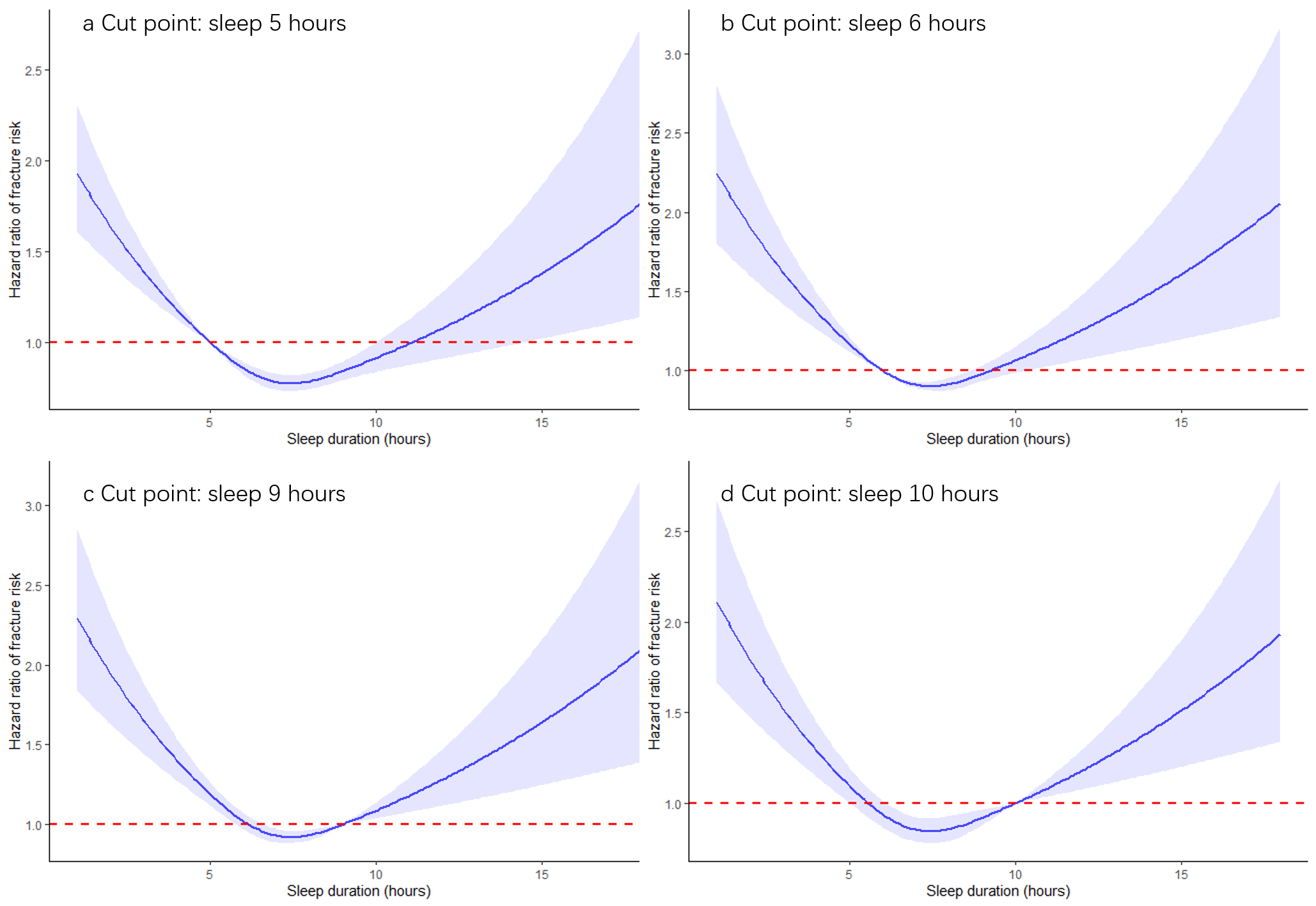


**Supplementary Figure 1** Observational association of sleep duration with fracture risk using a restricted cubic spline based on model 0 based on different cut points: (a) sleeping 5 hours per day; (b) sleeping 6 hours per day; (c) sleeping 9 hours per day; and (d) sleeping 10 hours per day. Hazard ratios are indicated by solid lines and the 95% confidence intervals by shaded areas. In all these analyses, models were adjusted for risk factors for fracture, including age, education, sex, smoking, alcohol consumption, physical activity, body mass index, and the use of glucocorticoid.
