# Supplementary material for: Observational and genetic evidence highlight the association of human sleep behaviors with the incidence of fracture
Source: Commun Biol. 2021 Nov 26;4:1339. doi: 10.1038/s42003-021-02861-0 (PMC8626439; doi:10.1038/s42003-021-02861-0)
Supplement: Supplementary file 3 — Description of Additional Supplementary Files [file 42003_2021_2861_MOESM3_ESM.pdf]

Description of Additional Supplementary Files

File name: Supplementary Data 1-4
Description:
Supplementary Data 1: Detailed information on the field ID and code for participants who were excluded in quality control in UK biobank.

Supplementary Data 2: Characteristics of the associations of instrumental variables for 4 sleep behaviors (i.e., chronotype, insomnia, snoring, and sleep duration) with fracture risk.

Supplementary Data 3: Detailed information on self-reported codes for five sleep behaviors (chronotype, insomnia, snoring, sleep duration, and excessive daytime sleepiness), the ICD-9 and ICD-10 codes for non-secondary fracture, and the codes for the corresponding diagnosis time in UK Biobank.

Supplementary Data 4: Detailed information on the definition and self-reported UK Biobank field codes for covariates which were adjusted in observational studies.

Supplementary Data 5: Source values for Figures 1 and 3.
