# Supplementary material for: Observational and genetic evidence highlight the association of human sleep behaviors with the incidence of fracture
Source: Commun Biol. 2021 Nov 26;4:1339. doi: 10.1038/s42003-021-02861-0 (PMC8626439; doi:10.1038/s42003-021-02861-0)
Supplement: Supplementary file 5 — Reporting Summary [file 42003_2021_2861_MOESM5_ESM.pdf]

nature portfolio

Corresponding author(s): Houfeng Zheng

Last updated by author(s): Sep 28, 2021

Reporting Summary

Nature Portfolio wishes to improve the reproducibility of the work that we publish. This form provides structure for consistency and transparency in reporting. For further information on Nature Portfolio policies, see our Editorial Policies and the Editorial Policy Checklist.

Statistics

For all statistical analyses, confirm that the following items are present in the figure legend, table legend, main text, or Methods section.

n/a	Confirmed	
☐	☒	The exact sample size (*n*) for each experimental group/condition, given as a discrete number and unit of measurement
☐	☒	A statement on whether measurements were taken from distinct samples or whether the same sample was measured repeatedly
☐	☒	The statistical test(s) used AND whether they are one- or two-sided<br>*Only common tests should be described solely by name; describe more complex techniques in the Methods section.*
☐	☒	A description of all covariates tested
☐	☒	A description of any assumptions or corrections, such as tests of normality and adjustment for multiple comparisons
☐	☒	A full description of the statistical parameters including central tendency (e.g. means) or other basic estimates (e.g. regression coefficient) AND variation (e.g. standard deviation) or associated estimates of uncertainty (e.g. confidence intervals)
☐	☒	For null hypothesis testing, the test statistic (e.g. *F*, *t*, *r*) with confidence intervals, effect sizes, degrees of freedom and *P* value noted<br>*Give P values as exact values whenever suitable.*
☒	☐	For Bayesian analysis, information on the choice of priors and Markov chain Monte Carlo settings
☐	☒	For hierarchical and complex designs, identification of the appropriate level for tests and full reporting of outcomes
☐	☒	Estimates of effect sizes (e.g. Cohen's *d*, Pearson's *r*), indicating how they were calculated

Our web collection on statistics for biologists contains articles on many of the points above.

Software and code

Policy information about availability of computer code

Data collection	All these statistical analyses were conducted in R version 4.0.2 (http://www.r-project.org/), and the PLINK2.0 software
Data analysis	All these statistical analyses were conducted in R version 4.0.2 (http://www.r-project.org/), and the PLINK2.0 software

For manuscripts utilizing custom algorithms or software that are central to the research but not yet described in published literature, software must be made available to editors and reviewers. We strongly encourage code deposition in a community repository (e.g. GitHub). See the Nature Portfolio guidelines for submitting code & software for further information.

Data

Policy information about availability of data

All manuscripts must include a data availability statement. This statement should provide the following information, where applicable:

- Accession codes, unique identifiers, or web links for publicly available datasets
- A description of any restrictions on data availability
- For clinical datasets or third party data, please ensure that the statement adheres to our policy

No additional data available. The code is available upon request.

Field-specific reporting

Please select the one below that is the best fit for your research. If you are not sure, read the appropriate sections before making your selection.

☒ Life sciences ☐ Behavioural & social sciences ☐ Ecological, evolutionary & environmental sciences

For a reference copy of the document with all sections, see nature.com/documents/nr-reporting-summary-flat.pdf

Life sciences study design

All studies must disclose on these points even when the disclosure is negative.

Sample size	The UK BioBank datasets encompass samples from over 500,000 individuals who participated in the UK Biobank. In our study, after quality control, the remained participants were included in final analyses (N = 421,761 for observational study; N = 282162 for one-sample mendelian randomization).
Data exclusions	We excluded 30,486 non-European participants to minimize the population stratification bias. Then, we excluded participants with potential comorbid diseases (i.e., rheumatoid arthritis, ulcerative colitis, multiple sclerosis, Crohn's disease, hyperthyroidism, or lupus erythematosus) (N = 28,657). Additionally, since this study is a prospective design, we also excluded participants who had suffered a fracture before baseline, and participants with secondary fractures (i.e., participants with follow-up care involving removal of the fracture plate and other internal fixation devices, pathological fractures, and the fracture of the bone in neoplastic disease), leaving 421,761 participants in the observational study. In the one-sample MR analysis, we further excluded 139,599 participants (Field ID 22021) who had a kinship with other participants.
Replication	All attempts at replication were successful.
Randomization	Randomization was not feasible for this study, since the study design was cohort. To control the influence of potential confounders, we performed the multivariable Cox regression. The basic model was adjusted for confounders, including age, sex, body mass index, education, smoking, alcohol consumption, physical activity, cognitive impairment, depression, and the use of glucocorticoid medication, benzodiazepines, and antidepressants (model 0), additional covariables such as BMD and falls were also included to set different models (mode1 1=model 0+BMD, model 2=model0+falls, and model 3=model 0+BMD+falls).
Blinding	Since we conducted the cohort study, the blinding was not suitable for this study.

Reporting for specific materials, systems and methods

We require information from authors about some types of materials, experimental systems and methods used in many studies. Here, indicate whether each material, system or method listed is relevant to your study. If you are not sure if a list item applies to your research, read the appropriate section before selecting a response.

Materials & experimental systems

n/a	Involved in the study	
☒	☐	Antibodies
☒	☐	Eukaryotic cell lines
☒	☐	Palaeontology and archaeology
☒	☐	Animals and other organisms
☐	☒	Human research participants
☒	☐	Clinical data
☒	☐	Dual use research of concern

Methods

n/a	Involved in the study	
☒	☐	ChIP-seq
☒	☐	Flow cytometry
☒	☐	MRI-based neuroimaging

Human research participants

Policy information about studies involving human research participants

Population characteristics	After excluding participants with missing data on the main covariates at baseline (n = 23,688), 13,002 out of the 398,073 participants (3.27 %) developed an incident fracture during a median of 7.96 years of follow-up. Please see the detailed information on Supplementary Table 6.
Recruitment	The recruitment of UK Biobank has been described elsewhere. Please see "UK biobank: an open access resource for identifying the causes of a wide range of complex diseases of middle and old age." for more details.
Ethics oversight	Ethics approval for the UK Biobank research was obtained from the North West Multicentre Research Ethical Committee, and all participants provided informed consent. This study was performed under generic ethical approval obtained by UK Biobank from the National Health Service National Research Ethics Service (approval letter ref 11/NW/0382, 17 June 2011)

Note that full information on the approval of the study protocol must also be provided in the manuscript.
